# Editorial Note: Retraction: The perception of Hungarian food by consumer segments according to food purchasing preferences based on primary research results

**DOI:** 10.1371/journal.pone.0330559

**Published:** 2025-08-19

**Authors:** The PLOS One Editors

This Retraction Notice [1] was erroneously published before PLOS concluded our assessment of an appeal. Materials provided with the appeal addressed the third point listed in the retraction notice [1] regarding the reporting of the results, but did not satisfactorily resolve the other concerns.

This notice updates the reasons underlying the retraction decision but does not affect the status of the article [2], which remains retracted.
